# SeSaMe PS Function: Functional Analysis of the Whole Metagenome Sequencing Data of the Arbuscular Mycorrhizal Fungi

**DOI:** 10.1016/j.gpb.2018.07.011

**Published:** 2020-12-18

**Authors:** Jee Eun Kang, Antonio Ciampi, Mohamed Hijri

**Affiliations:** 1 Institut de Recherche en Biologie Végétale, Département de Sciences Biologiques, Université de Montréal, QC H1X 2B2, Canada; 2 Department of Epidemiology, Biostatistics and Occupational Health, McGill University, Montréal, QC H3A 1A2, Canada

**Keywords:** SeSaMe, Spore-associated symbiotic microbes, Position-specific function, Outlier, Metagenome

## Abstract

In this study, we introduce a novel bioinformatics program, **Spore-associated Symbiotic Microbes Position-specific Function** (**SeSaMe** PS Function), for position-specific functional analysis of short sequences derived from **metagenome** sequencing data of the arbuscular mycorrhizal fungi. The unique advantage of the program lies in databases created based on genus-specific sequence properties derived from protein secondary structure, namely amino acid usages, codon usages, and codon contexts of 3-codon DNA 9-mers. SeSaMe PS Function searches a query sequence against reference sequence database, identifies 3-codon DNA 9-mers with structural roles, and creates a comparative dataset containing the codon usage biases of the 3-codon DNA 9-mers from 54 bacterial and fungal genera. The program applies correlation principal component analysis in conjunction with K-means clustering method to the comparative dataset. 3-codon DNA 9-mers clustered as a sole member or with only a few members are often structurally and functionally distinctive sites that provide useful insights into important molecular interactions. The program provides a versatile means for studying functions of short sequences from metagenome sequencing and has a wide spectrum of applications. SeSaMe PS Function is freely accessible at www.fungalsesame.org.

## Introduction

Arbuscular mycorrhizal fungi (AMF) are plant root colonizing symbiotic microorganisms that promote plant growth and improve soil quality [1], [2], [3]. AMF increase the effectiveness of phytoremediation and improve crop yields in agroecosystems [1], [4], [5], [6], [7], [8], [9], [10]. Despite the importance of AMF, their genetics is poorly understood, due in large part to their coenocytic multinucleate nature and strict symbiotic partnership with plants [11]. A number of studies reported strong evidence that AMF interact closely — tightly adhering to the surface or in the interior of mycelia and spores — or loosely with a myriad of microorganisms covering major bacterial and fungal taxa [6], [12], [13], [14], [15], [16]. These microorganisms can be removed from AMF by using cocktails of antibiotics in axenic cultivation systems [17]. Yet, only few AMF taxa are able to be cured and cultivated *in vitro,* and most successful isolates in such systems mainly belong to the genus *Rhizophagus* [18]. Given that the majority of AMF have not been successfully cultured axenically, it is possible that AMF may be meta-organisms, inseparable from their bacterial and fungal partners.

Whole genome sequencing (WGS) of AMF taxa has been achieved exclusively from those grown *in vitro*. Although they provide important insights into AMF genetics, they have limitations in serving as reference genome due to large intra- and inter-isolate genome variations [19], [20]. Furthermore, sequence analysis of the WGS of AMF taxa grown *in vivo*, typically in a pot culture with a host plant, can be challenging because the sequencing data contain a large proportion of sequences belonging to AMF-associated microorganisms; the WGS data of AMF represent a complex metagenome [16], [21]. However, they provide invaluable information about the associated microbial community because a great majority of the associated microorganisms cannot be cultured in laboratory conditions. Taxonomic classification of the whole metagenome sequencing (WMS) data is essential for studying AMF genomics and their interactions with the associated microorganisms. We introduced the bioinformatics program, Spore-associated Symbiotic Microbes (SeSaMe), for taxonomic classification of the WMS of AMF [22]. In this study, we introduce a novel bioinformatics program — SeSaMe Position-specific Function (SeSaMe PS Function). It predicts important position-specific functional sites in a query sequence, based on amino acid usages, codon usages, and codon contexts of 3-codon DNA 9-mers derived from protein secondary structures extracted from Protein Data Bank (PDB) (https://www.rcsb.org/) [23].

Previous studies have documented the multiple regulatory roles of codon usage and codon context in transcription and translation (*e.g.*, regulation of gene expression, diversification of gene products, translational efficiency and accuracy, and protein degradation efficiency) [24], [25], [26], [27], [28], [29], [30]. Several studies have emphasized the regulatory roles of codon usage and codon context of multiple consecutive codons [25], [29], [30]. In addition, synonymous codons are believed to be a key factor in determining the active folding state of a gene product in response to environmental changes. One study has shown that a gene with multiple synonymous mutations produces a protein with increased tolerance to abiotic stresses [31]. Moreover, non-optimal codons serve specific roles in regulating circadian rhythms in response to changes of environmental conditions [32], [33]. Therefore, codon usage and codon context must have been playing important roles in the adaptation of microorganisms to abiotic stresses [34], [35]. We are beginning to scratch the surface of the regulatory roles of codon usage and codon context, and these studies appear to be just a tip of iceberg.

The main variable of the program — trimer usage bias — takes usages and contexts of both amino acids and nucleotides into consideration; it is the product of amino acid usage and 3-codon usage of 3-codon DNA 9-mer. Generally, trimer usage bias has a broad range of variations among taxonomic groups but low variations among microorganisms belonging to the same taxonomic group. Trimer usage bias reflects the important attributes of multiple consecutive codons. Codon composition (*i.e.*, codon context of three consecutive codons) is an important determinant of properties of RNA structures that plays key roles in regulating gene expression. Codon usage is associated with pauses in translation and determines biochemical properties of gene products. Both of the attributes affect protein folding.

**Figure 1:**
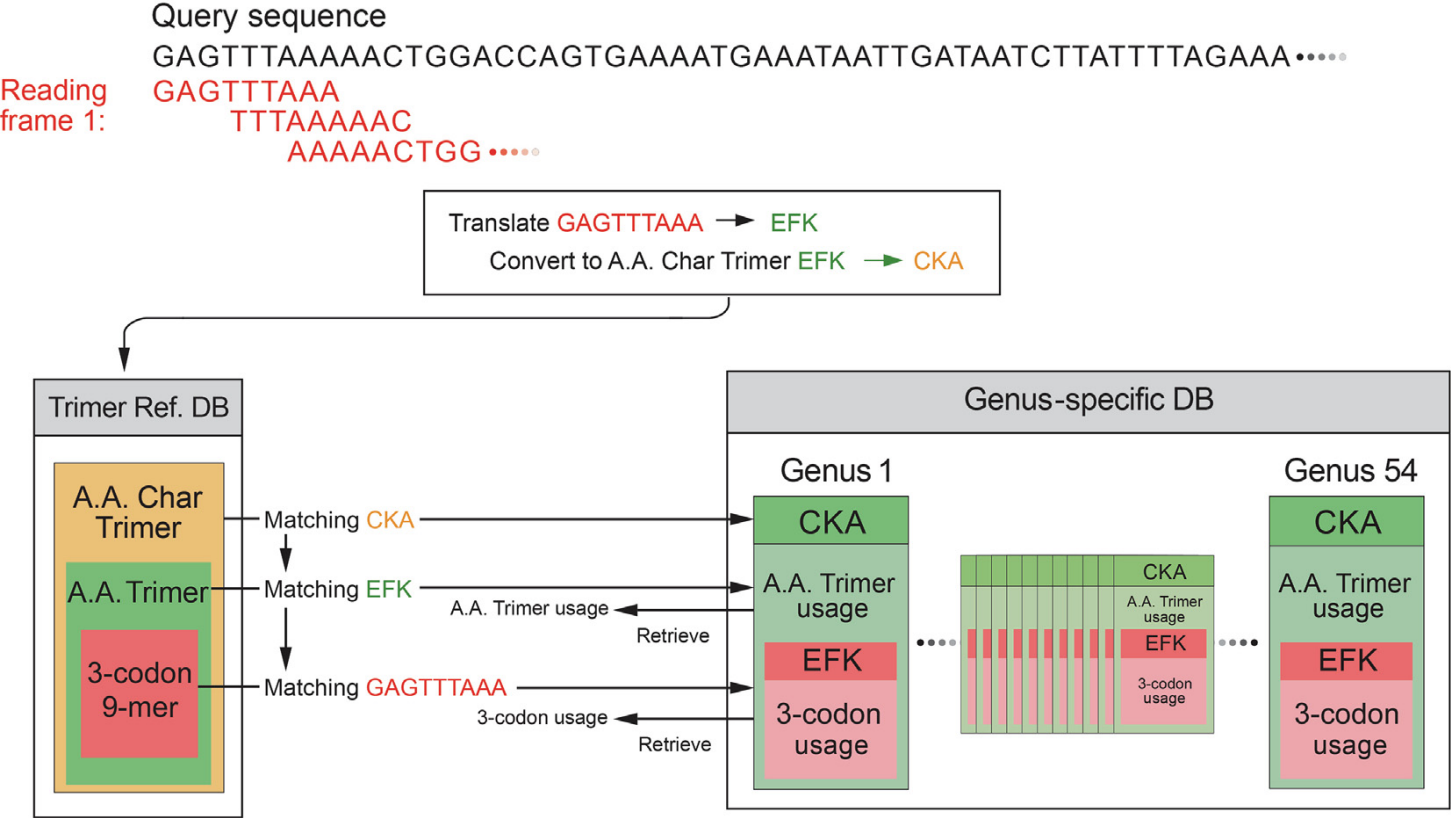
**Dynamic creation of a comparative dataset per query sequence** SeSaMe PS Function uses a query sequence to search matching A.A. Char Trimers, A.A. Trimers, and 3-codon DNA 9-mers in Trimer Ref. DB, and retrieves the A.A. Trimer usages of the matching A.A. Trimers and the 3-codon usages of the matching 3-codon DNA 9-mers from 54 genus-specific DBs. It calculates the trimer usage biases of the matching 3-codon DNA 9-mers, and generates a comparative dataset for the query sequence. SeSaMe PS Function, Spore-associated Symbiotic Microbes Position-specific Function; A.A. Trimer, amino acid trimer; A.A. Char Trimer, amino acid characteristic trimer; Trimer Ref. DB, trimer reference sequence database; genus-specific DB, genus-specific usage bias database.

SeSaMe PS Function identifies 3-codon DNA 9-mers with structural roles in a query sequence, and creates a comparative dataset based on their trimer usage biases that are retrieved from 54 genus-specific usage bias databases (genus-specific DBs) (Figure 1). SeSaMe PS Function applies correlation Principal Component Analysis (PCA) in conjunction with K-means clustering method (PCA-Kmeans) to the comparative dataset. It enables users to identify 3-codon DNA 9-mers with distinctive characteristics: outliers. Outliers are often important position-specific functional sites that provide useful insights into molecular interactions.

In this study, we analyzed one example sequence to demonstrate how to use the program for studying the structure and the function of a query sequence: one of the program’s various applications. The program helped to identify the outliers with potentially important functions. Existing bioinformatics programs predicted that most of the outliers belonged to stem-loops, stems, and stem transitions in RNA structures [36]. Some of the outliers were matched to elements that play roles in promotor regions or in *cis*-regulatory mechanisms [37], [38], [39]. Other bioinformatics programs predicted that the example sequence may bind to DNA/RNA [23], [40]. These results suggest that the outliers may contribute to binding activities in undiscovered mechanisms that may have attributes similar to *cis*-regulatory mechanisms.

A majority of existing bioinformatics tools for position-specific sequence annotation rely on sequence alignments, which have low sensitivity toward hypervariable sequence motifs with flexible structures and various functions. Although they provide important information about a query sequence, their usage is limited to a particular set of motifs with known functions. In contrast, SeSaMe PS Function employs PCA to identify outliers based on internal structure of a comparative dataset that contains usage information of structural units of a query sequence measured in 54 genera. Therefore, it may reveal important molecular interaction sites not only in known but also in undiscovered mechanisms. It has been only several decades since advances have been made in molecular biology. Therefore, it is believed that only a small fraction of mechanisms in biological systems have been discovered. SeSaMe PS Function provides a useful tool for studying unknown functions of short sequences from metagenome sequencing data. It is freely accessible at www.fungalsesame.org.

## Method

### Database design and comparative dataset creation

The databases were originally created for the metagenome taxonomic classifier — SeSaMe, and then incorporated into SeSaMe PS Function [22]. While NCBI offered a large number of completely sequenced bacterial genomes, only a small number of fungal genomes were completely sequenced. The completely sequenced genomes of 444 bacteria and 11 fungi, known to be present in soil, were downloaded and assigned into 45 bacterial and 9 fungal genera, respectively. CDS database per genus was created based on CDS lists provided by NCBI, JGI, or Tisserant et al. [19].

**Figure 2:**
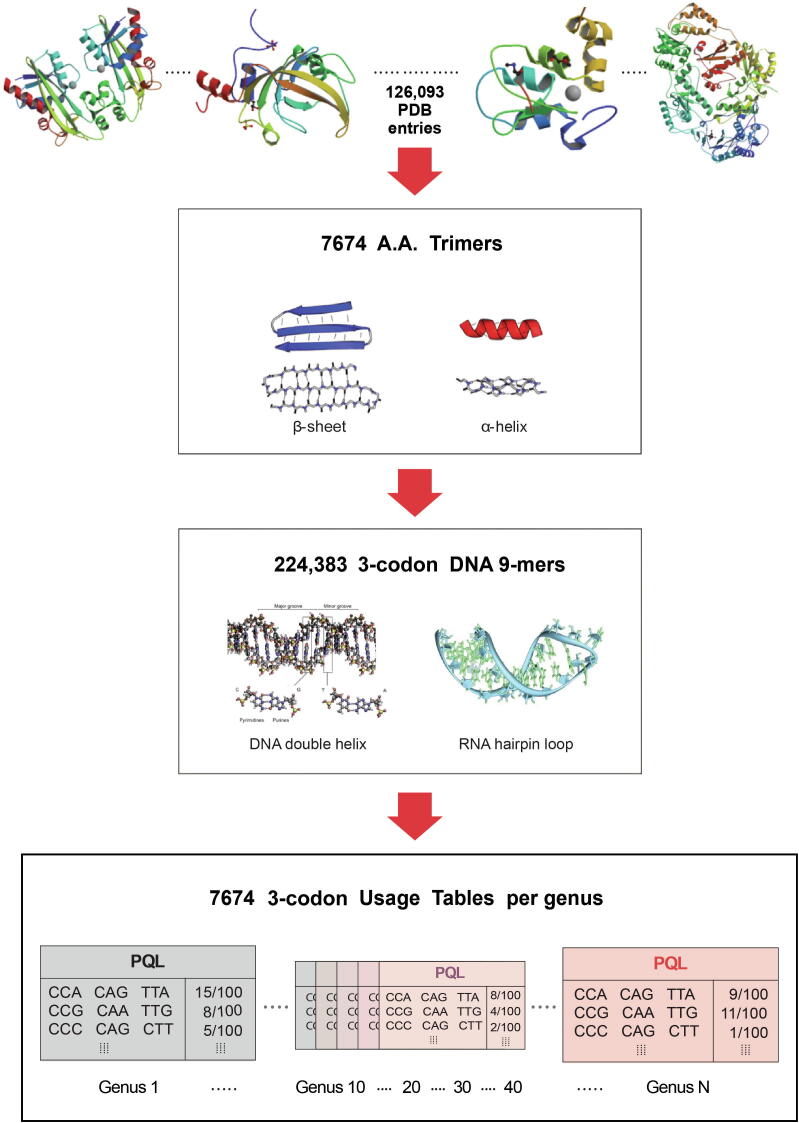
**Database design** A large number of PDB entry files were processed to extract 7674 A.A. Trimers — subunits of protein secondary structures. A table of 3-codon usage was created per A.A. Trimer and per genus in genus-specific DB. The PDB IDs of the protein structures shown in the top panels from left are 2ZTI, 3DWH, 2VSL, and 3DRP [41], [42], [43], [44]. Images of the protein secondary structures in the first rectangle and the nucleotide structures in the second rectangle are obtained from https://en.wikipedia.org/wiki/Alpha_helix and https://en.wikipedia.org/wiki/RNA, respectively.

The program consists of two types of databases and a PCA-Kmeans method. 126,093 structure files were downloaded from PDB. 7674 amino acid trimers (A.A. Trimers) were selected among protein secondary structures from PDB, and then assigned to the sequence variable — A.A. Trimer in the trimer reference sequence database (Trimer Ref. DB) (Figure 2) [41], [42], [43], [44]. Amino acid characteristic (A.A. Char) is defined as a group of amino acid(s) with similar property(s), and consists of 12 groups: A [Lysine (K), Arginine (R)], B [Histidine (H)], C [Aspartic acid (D), Glutamic acid (E)], D [Serine (S), Threonine (T)], E [Asparagine (N), Glutamine (Q)], F [Cysteine (C)], G [Glycine (G)], H [Proline (P)], I [Methionine (M)], J [Alanine (A), Isoleucine (I), Leucine (L), Valine (V)], K [Phenylalanine (F), Tryptophan (W), Tyrosine (Y)], and L (stop codons). Trimer Ref. DB consists of three sequence variables that form a three-level hierarchy: amino acid characteristic trimer (A.A. Char Trimer), A.A. Trimer, and 3-codon DNA 9-mer (Figure 1).

Genus-specific usage bias database (genus-specific DB) contains the numerical variables — A.A. Trimer usage of A.A. Trimer and 3-codon usage of 3-codon DNA 9-mer. The main numerical variable, trimer usage bias, is calculated by multiplying A.A. Trimer usage by 3-codon usage. There are 54 genus-specific DBs where each genus-specific DB consists of 1296 A.A. Trimer Usage Tables and 7674 3-codon Usage Tables created based on the CDS database (Figure 2).

For each reading frame of a query sequence, the program uses a query sequence to search against Trimer Ref. DB, identifying matching A.A. Char Trimers, A.A. Trimers, and 3-codon DNA 9-mers. It retrieves the trimer usage biases of the matching 3-codon DNA 9-mers from 54 Genus-specific DBs, and creates a comparative dataset of 54 genera (Figure 1). The input matrix to the correlation PCA method is the comparative dataset with 54 genera in rows (observations) and the matching 3-codon DNA 9-mers in columns. The input matrix will be called hereafter Z (I × J).

### Annotation for catalytic and allosteric sites

According to Catalytic Site Atlas and Allosteric Database, A.A. Trimers were divided into four subgroups based on the property of their second amino acids: catalytic site (CSA), allosteric site (ASD), both CSA and ASD (BothCA), and none of them (None) [45], [46]. An A.A. Trimer in CSA, ASD, or BothCA groups was annotated with the list of functions of PDB molecules that contained the A.A. Trimer. This feature is for making inferences about functionality not of a query sequence but of its A.A. Trimers.

### Implementation of the correlation PCA-Kmeans method

The correlation PCA method was implemented based on the method reported by Abdi et al. [47], which provides important definitions and multiple examples to help readers understand the concepts underlying PCA [47]. Interpretation of the result from SeSaMe PS Function also relies on Abdi et al. [47] because eigenvalue decomposition is mathematically closely related to singular value decomposition and has similar underlying concepts. Pearson's correlation method is applied to the centered Z and produces a correlation matrix X (J × J). Eigenvalue decomposition is applied to X and produces components. V is an eigenvector matrix with J × J dimensions and is also called a loading matrix.

#### Loadings: elements of the loading eigenvector matrix V

The program calculates an eigenvector matrix V. Loading is defined as the element of V. V has matching 3-codon DNA 9-mers in rows and the same number of components in columns. The program examines loadings on components whose sum accounts for 80% of inertia (80% components) in addition to loadings on the first principal component and the second component (the First/Second components) [47]. The program creates two different input matrices based on V, called L1 and L2. They have the same number of 3-codon DNA 9-mers in the rows. L1 has 80% components in columns while L2 has the First/Second components in columns. The program separately applies the K-means clustering method (default k = 13) to L1 and L2.

#### Taxon scores of 54 genera in component spaces

The program calculates taxon scores of 54 genera observations. Taxon score matrix (I × J) results from multiplying centered Z by V. Inertia of a component is defined as a sum of squared taxon scores in corresponding component column [47]**.** The program creates two matrices based on taxon score matrix called T1 and T2. They have 54 genera observations in rows. T1 has 80% components in columns, while T2 has the First/Second components in columns. The program separately applies the K-means clustering method (default k = 10) to T1 and T2.

### Program availability

The program was implemented in Java programming language (Java8). We used the Pearson's correlation, the eigenvalue decomposition, and the K-means clustering methods in the Apache Commons Math3 library (3.3). The program requires the Apache Commons Math3 (3.3) and IO (2.4) libraries (www.apache.org). The program has been made to run on Linux/Unix operating systems, packaged into an executable Java JAR file, and tested and confirmed to work on Linux system — CentOS Linux 7 (www.centos.org). The program that is being introduced in this study is version 1 and was implemented with the correlation PCA only. The program (version 2) was implemented both with the covariance PCA and with the correlation PCA. They have been used at the Biodiversity Center, Institut de Recherche en Biologie Végétale, Département de Sciences Biologiques, Université de Montréal. They are freely accessible at www.fungalsesame.org. There are no restrictions for using the programs by academic or non-academic organizations as long as a user complies with the license agreement.

### Input, output, and options

The program has a command-line interface. Input files should contain DNA sequence(s) in fasta format. It requires a command-line argument, input file path. SeSaMe PS Function produces three different types of outputs per query sequence. One is the standard PCA output: the sequence information of matching 3-codon DNA 9-mers, the percentage of an explained inertia by a component, and the contribution of an observation to a component [47]. Another is the loading cluster output with the loading information. 3-codon DNA 9-mers are annotated with subgroups — CSA/ASD/BothCA/None and the functions of PDB molecules. The other is the genus cluster output with the taxon scores. It should be noted that the cluster result is different for every run, because the K-means clustering method in the Apache Commons Math library randomly chooses initial centers for multiple iterations to decrease chances of poor clustering.

SeSaMe PS Function version 1 and version 2 have an option to specify the k parameter in the K-means clustering method both for genus clusters and for loading clusters (*e.g.*, 11_15). The program version 2 has an additional option called “auto”. If a user wants to run SeSaMe PS Function for a large number of query sequences with varying lengths, he can use the prefix “auto” to set the k parameter for loading clusters according to a simple equation: the number of matching 3-codon DNA 9-mers divided by a user specified number. For example, if the user gives the following option “auto_14_8”, it will automatically set one eighth of the number of matching 3-codon DNA 9-mers as the k parameter for loading clusters while it will set 14 as the k parameter for genus clusters. A suitable k value may vary widely depending on the length and the complexity of a query sequence. User can supply the option following the input file path (*e.g.*, /home/input-file auto_14_8).

### Demonstration of the program usage

#### Selection of the example sequence

We selected 25 correctly predicted sequences out of 100 AMF CDS test sequences that were used for evaluating the accuracy of the metagenome taxonomic classifier, SeSaMe [22]. From 25 sequences, we selected one example sequence that had the largest number of 3-codon DNA 9-mers where AMF had the highest trimer usage bias among 54 genera. The example query sequence is TGAGTTTAAAAACTGGACCAGTGAAAATGAAATAATTGATAATCTTATTTTAGAAATGCAATTAAAAATTAATAGTACATATGATAAAATAGTTGAATGGATACCATACAATCAGTTTATTAACATTAACGAAATAGGAAAAGTTGGTGATAATACTGCTGTATATTCAGCAATATGGAAAAATGGTCCACTATATTATAGAAAGAAATGGATAAGGAAATCCAATGAAAAAGTTGTATTAAATTACTTAACATTAGATATTAAGGAATT.

#### Outlier's unique pattern of the trimer usage bias and of the 3-codon usage

Landscape pattern is the comparison of 54 genera based either on the trimer usage bias or on the 3-codon usage of a 3-codon DNA 9-mer. It provides an accurate way to estimate the relative measure of the usage information across 54 genera. In this study, we abbreviate 3-codon DNA 9-mer according to the order of its position in DNA sequence and its A.A. Trimer (Table S1). For example, AATACTGCT is the 51st matching 3-codon DNA 9-mer and encodes for the amino acids NTA. Because the program is zero-based, its abbreviation is 50 NTA. Graphs showing the landscape patterns of 3-codon usages and of trimer usage biases retrieved from 54 genera were generated for 17 EMQ and 67 KKW and for 18 MQL and 3 NWT, respectively.

#### Comparison of the frequencies of a nucleotide among 13 loading clusters

We counted the frequencies of the nucleotide — adenine (A) — in each of the individual 3-codon DNA 9-mers and applied a one-way ANOVA test to compare the means among 13 clusters. We repeated the same process for the nucleotides cytosine (C), guanine (G), and thymine (T).

#### Comparison between the trimer usage bias and the 3-codon usage in functional segment

We assigned matching 3-codon DNA 9-mers into functional segments (FSs) based on the loading clusters with 80% components and based on the prediction result of the protein secondary structure from a bioinformatics tool — SCRATCH [48].

We created two matrices per FS: one was based on the 3-codon usage, and the other was based on the trimer usage bias. Each matrix consisted of the usage information of the matching 3-codon DNA 9-mers retrieved from 54 genera; it had the 3-codon DNA 9-mers of an FS in rows and the 54 genera in columns. After centering each matrix, we applied Pearson's correlation to the matrix to yield a correlation matrix (I × I), and calculated the mean of the correlations per pair of taxonomic groups — Clostridia, Bacilli, Oscillatoriophycideae, Nostocales, Acidobacteria, Alphaproteobacteria, Betaproteobacteria, Deltaproteobacteria, Gammaproteobacteria, AMF, Agaricomycotina, and Pezizomycotina. From the mean of the correlations of a pair of genera belonging to the same taxonomic group in each FS, we calculated the mean and the standard deviation per taxonomic group. In the same way, we calculated the mean of the correlations for pairs of taxonomic groups — Firmicutes, Cyanobacteria, Proteobacteria, Actinobacteria, AMF, a group of 7 Dikarya, and *Phanerochaete* in each FS.

## Results

### Loading clusters

The example sequence had 270 bp. When we ran the metagenome taxonomic classifier — SeSaMe — with the example sequence, it had the highest trimer usage probability score in the 2nd reading frame translation [22]. It had 87 matching 3-codon DNA 9-mers in the 2nd reading frame translation. The PCA method applied to the comparative dataset showed that 51 components represented 80% components, while the First/Second components explained approximately 29% of total inertia.

The K-means clustering method (*k* = 13) applied to the loadings of 80% components identified outliers, 14 3-codon DNA 9-mers. One major cluster had 73 members. 12 clusters had 14 outliers: 10 clusters had a sole member (50 NTA, 63 LYY, 72 KSN, 4 WTS, 69 WIR, 73 SNE, 24 STY, 30 VEW, 80 NYL, and 51 TAV), while 2 clusters had 2 members (33 IPY and 61 GPL, and 39 INI and 86 IKE).

**Supplementary figure S1:**
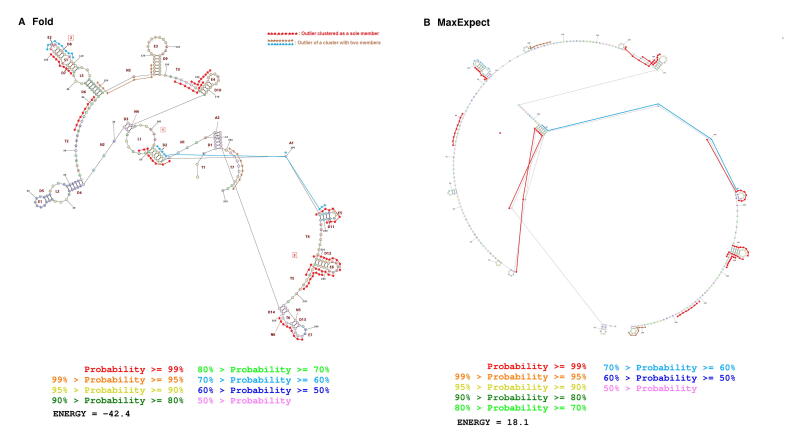
**Outliers in the predicted mRNA secondary structures**. The structures were generated by the bioinformatics program–RNAstructure (https://rna.urmc.rochester.edu/RNAstructureWeb/Servers/Predict1/Predict1.html). A. The example DNA sequence with T replaced with U was submitted as RNA. The secondary structure was predicted based on the algorithm called Fold. B. The example DNA sequence was submitted as DNA. The secondary structure was predicted based on the algorithm called MaxExpect.

Structural homology search in PDB and inference of DNA-binding residues in DRNApred suggested that the example sequence may be a DNA/RNA binding protein [23], [40]. We used the outliers to search publicly available bioinformatics databases containing DNA motifs with known functions. RSAT indicated that the outlier and its adjacent 3-codon DNA 9-mer (*i.e.*, 4 WTS and 3 NWT) were matched to motifs involved in *cis*-regulatory mechanisms, one in the + strand and the other in the – strand [37]. BPROM (Prediction of bacterial promoters) predicted that the outliers 30 VEW and 33 IPY were promoter-related elements [38]. GPMiner indicated that three outliers (4 WTS, 33 IPY, and 61 GPL) were matched to statistically significant over-represented oligonucleotides in the promoter region [39]. RNA structure prediction tools predicted that most outliers formed stem-loops, stems, and transition routes to stem in RNA structure of the example sequence (Figure S1) [36]. A large number of studies have documented stem-loop and stem structures in RNAs as important regulatory sites and binding sites [49], [50]. Considering that we are just beginning to understand the regulatory roles of codon usage and codon context, considerable portions of outliers and their adjacent 3-codon DNA 9-mers identified by the program may serve important roles in undiscovered mechanisms.

**Figure 3:**
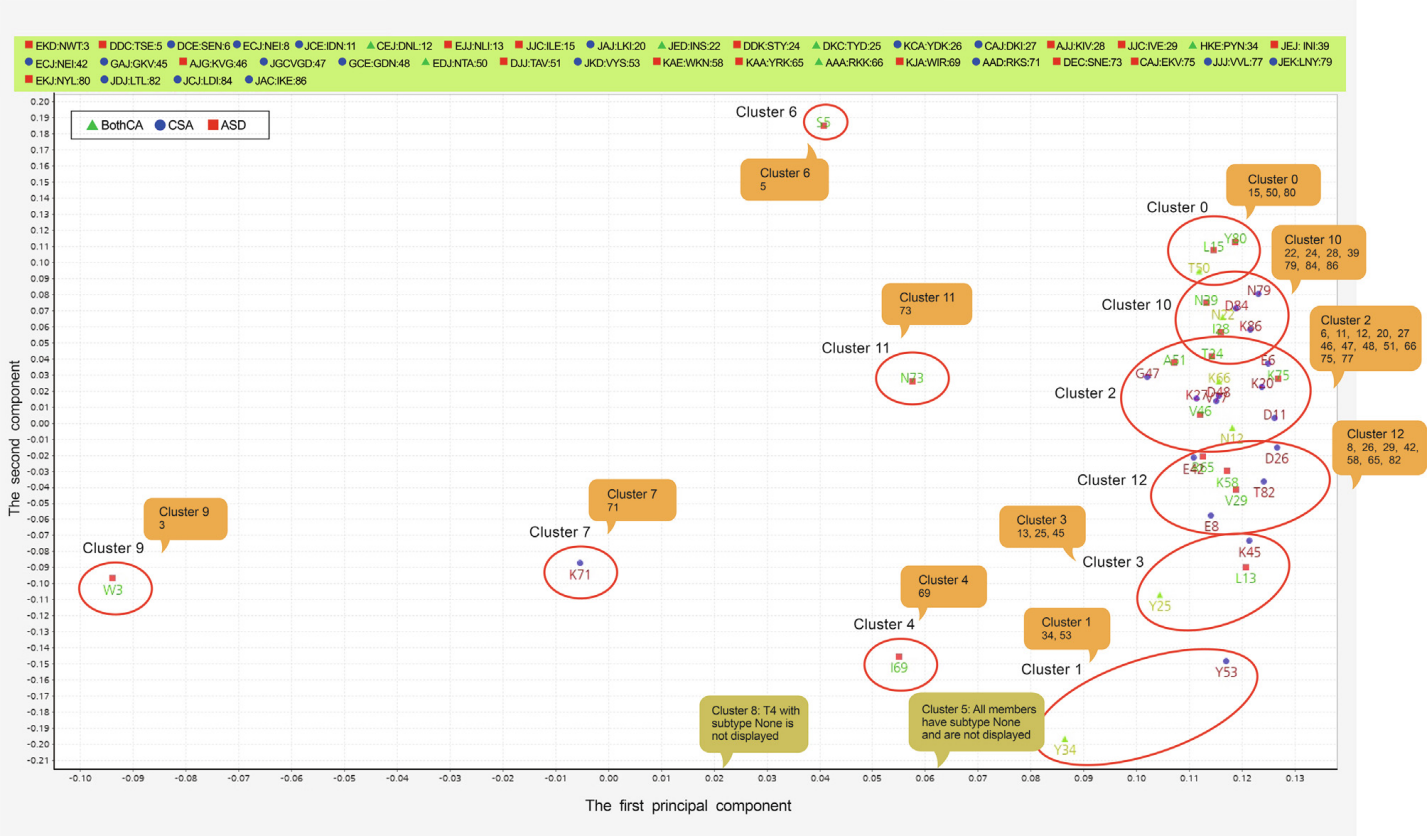
**Loading clusters of the example sequence** The figure shows elements of the loading matrix V on the space of the First/Second components. A name of 3-codon DNA 9-mer is abbreviated to the second amino acid of its A.A. Trimer. For example, 3-codon DNA 9-mer (AACTGGACC) that encodes the A.A. Trimer NWT is abbreviated to W (Table S1). The number following the abbreviation indicates the order of its position in the example sequence. The number in the colored box is the number following the abbreviation of a 3-codon DNA 9-mer. For example, 22 under cluster 10 in the box stands for ATTAATAGT that encodes the A.A. Trimer INS whose order of the position is 22. CSA, catalytic site; ASD, allosteric site; BothCA, both CSA and ASD; None, none of these sites.

The loading clusters with the First/Second components based on the trimer usage bias are shown in Table S1. It should be noted that Table S1 indicates the 3-codon usages for comparison purpose, which will be discussed in another section. The loadings of 3-codon DNA 9-mers with the catalytic or allosteric site in the second amino acid were plotted on the space of the First/Second components (Figure 3). A majority of 3-codon DNA 9-mers where Firmicutes, Cyanobacteria, *Rickettsia,* or AMF had the highest 3-codon usage were aggregately located on the far-right side (Figure 3). In contrast, those where Deltaproteobacteria, Gammaproteobacteria, or Actinobacteria had the highest 3-codon usage were dispersed across the left side and the middle of the graph. For example, 3 NWT where *Kocuria* had the highest value was located on the far-left side (Table S1).

### Genus clusters

**Figure 4:**
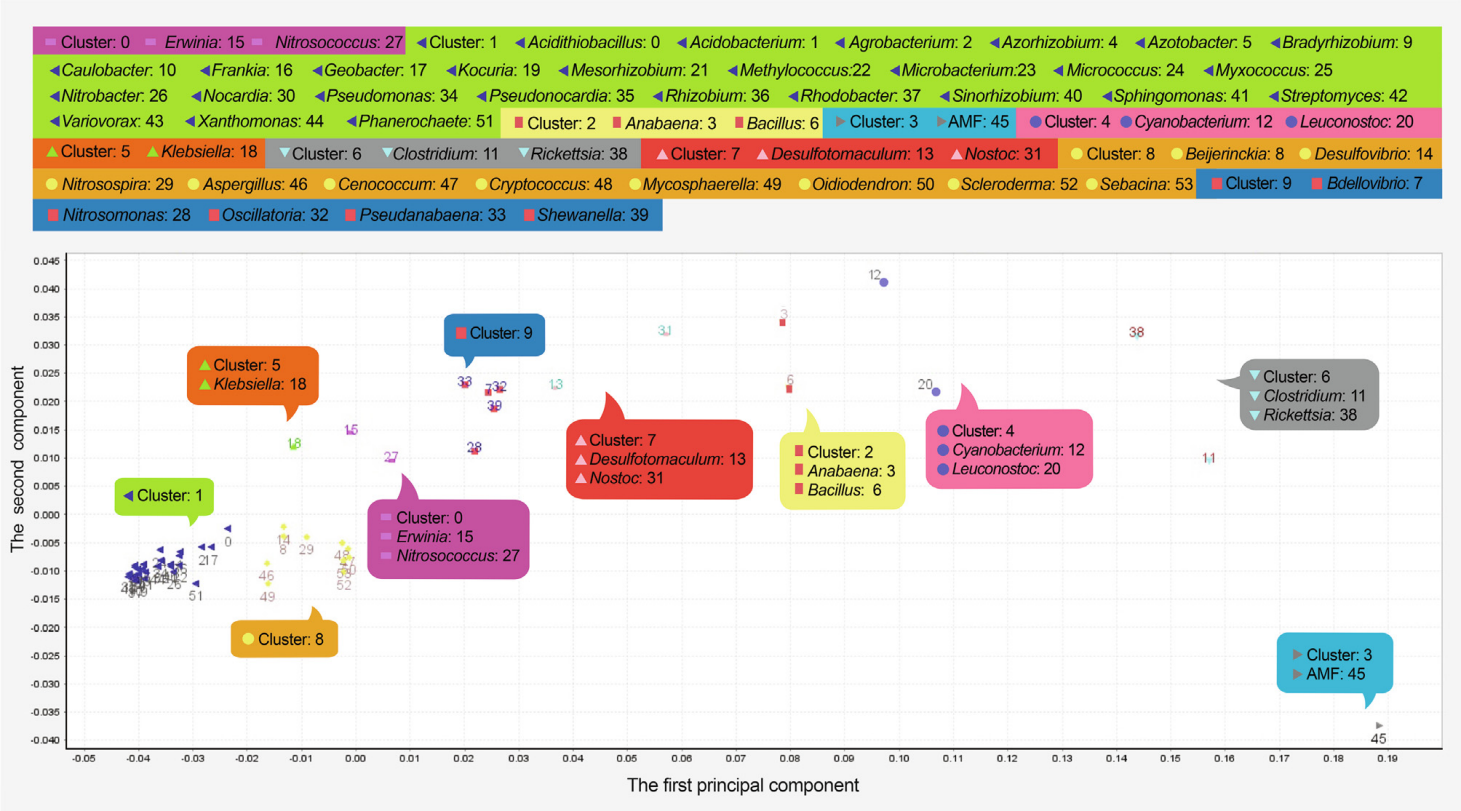
**Genus clusters of the example sequence** Taxon scores of 54 genera are plotted on the space of the First/Second components. Because the program is zero-based, individual of 54 genera is labeled with a number from 0 to 53.

The genus clusters based on 80% components indicated that genera with close phylogenetic relationships were assigned to the same cluster. In the scatter plot of taxon scorers on the space of First/Second components, Firmicutes, Cyanobacteria, *Rickettsia*, and AMF that frequently had high trimer usage biases were located on the right, while most members of Actinobacteria and Proteobacteria (cluster 1) that frequently had low values were located on the far-left side (Figure 4).

### Outlier's unique landscape pattern of trimer usage bias and of 3-codon usage

For each of the 3-codon DNA 9-mers in loading clusters with the First/Second components, we ranked 54 genera in order of decreasing 3-codon usages. We then ranked the 3-codon DNA 9-mers in each subgroup (CSA/ASD/BothCA/None) of the clusters based on a maximum of the 3-codon usages (Table S1). The mean of the maxima was 0.256. AMF, *Clostridium*, and *Rickettsia* frequently had the maximum.

**Figure 5:**
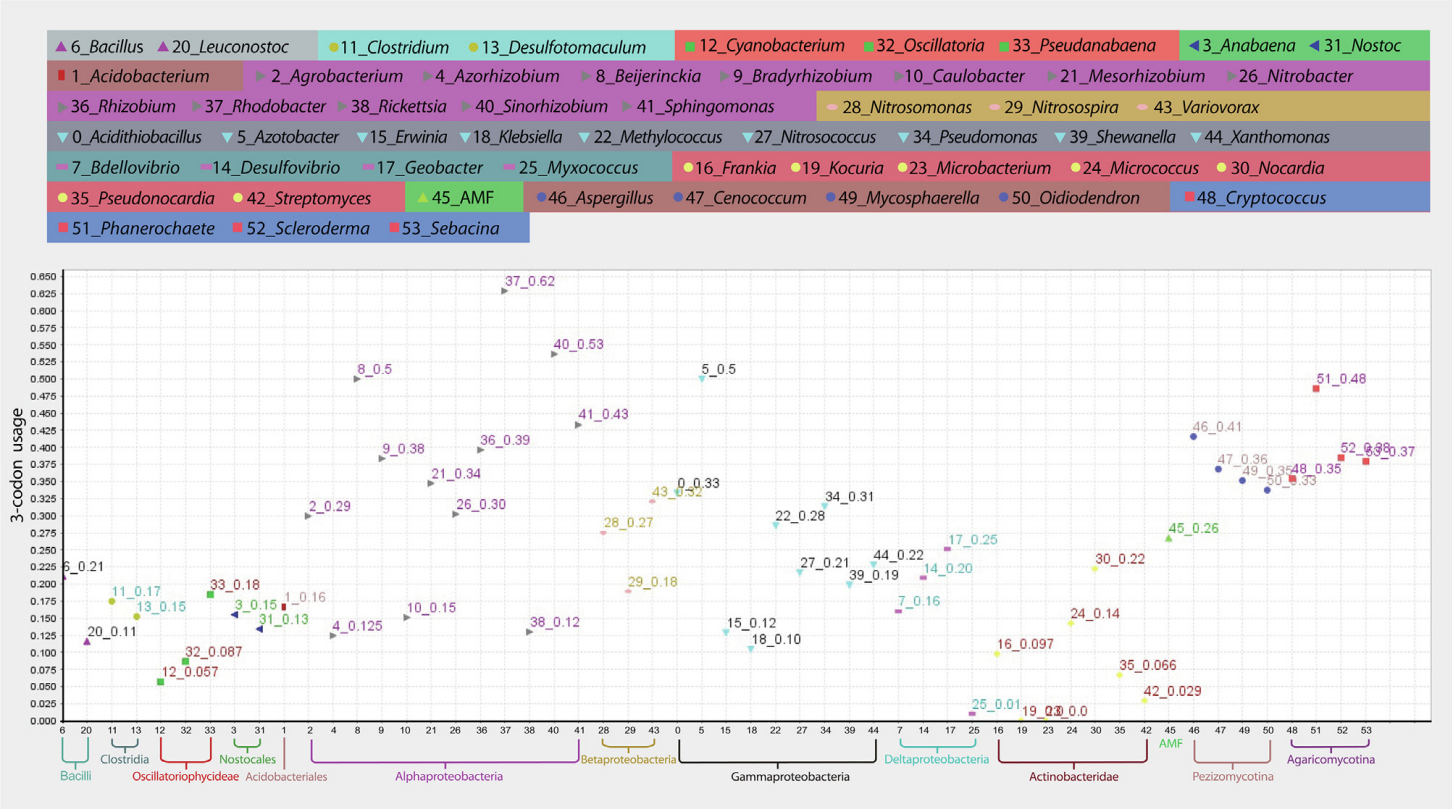
**Landscape pattern of the 3-codon usage of 67-AAK-KKW-AAGAAATGG** 54 genera are arranged into 13 taxonomic groups that are coded in different colors. Because the program is zero-based, individual of 54 genera is labeled with a number from 0 to 53.

**Supplementary figure S2:**
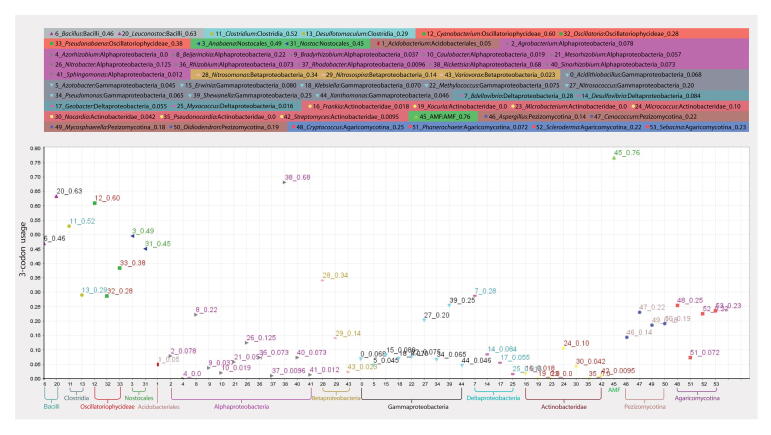
**Landscape pattern of the 3-codon usage of 17-CIE-EMQ-GAAATGCAA**. 54 genera are arranged into 13 taxonomic groups. Because the program is zero-based, individual of 54 genera is labeled with a number from 0 to 53.

**Supplementary figure S3:**
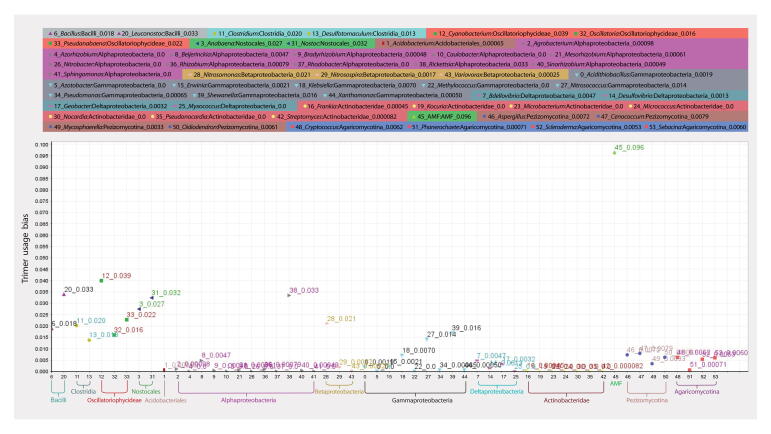
**Landscape pattern of the trimer usage bias of 18-IEJ-MQL-ATGCAATTA**. 54 genera are arranged into 13 taxonomic groups. Because the program is zero-based, individual of 54 genera is labeled with a number from 0 to 53.

**Supplementary figure S4:**
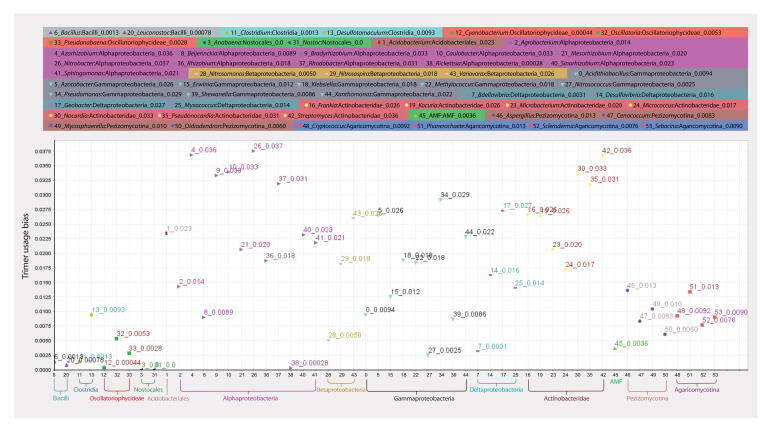
**Landscape pattern of the trimer usage bias of 3-EKD-NWT-AACTGGACC**. 54 genera are arranged into 13 taxonomic groups. Because the program is zero-based, individual of 54 genera is labeled with a number from 0 to 53.

Most of 3-codon DNA 9-mers in the major cluster demonstrated similar landscape patterns of the 3-codon usage and of the trimer usage bias. For example, 17-CIE-EMQ-GAAATGCAA and 18-IEJ-MQL-ATGCAATTA had the frequently demonstrated landscape pattern (Figures S2 and S3**)**. Outliers had a unique landscape pattern; for example, genera belonging to Dikarya had a higher value than AMF both in 67-AAK-KKW-AAGAAATGG and 3-EKD-NWT-AACTGGACC (Figure 5, Figure S4).

### Comparison of the frequencies of a nucleotide among 13 loading clusters

One-way ANOVA tests showed that the means of the frequencies of G and C in each of the individual 3-codon DNA 9-mers were significantly different among 13 clusters; F-statistics and *P* values of A, T, G, and C among 13 clusters were 0.69 (0.76), 1.26 (0.26), 1.91 (0.047), and 3.09 (0.0014), respectively.

### Comparison between the trimer usage bias and the 3-codon usage in FS

**Supplementary figure S5:**
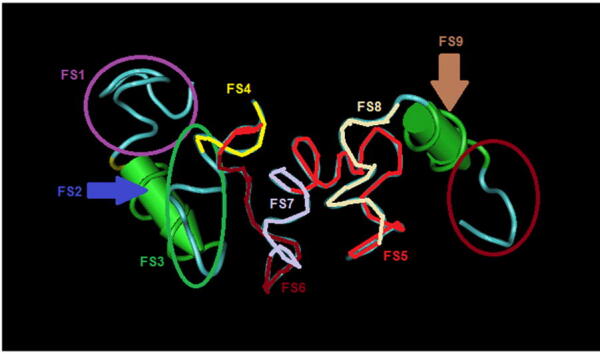
**FSs of the predicted protein tertiary structure**. The structure was predicted by the bioinformatics program–SCRATCH (http://scratch.proteomics.ics.uci.edu/). The PDB file format was converted to Cn3D format by another bioinformatics program–Vast (https://www.ncbi.nlm.nih.gov/Structure/VAST/vastsearch.html). The 3-dimensional structure was viewed by Cn3D (https://www.ncbi.nlm.nih.gov/Structure/CN3D/cn3d.shtml).

We merged some of the outliers in 12 clusters according to their proximity in the example sequence, which produced 8 groups. The merged outliers were 50 NTA with 51 TAV, 33 IPY and 61 GPL with 63 LYY, as well as 69 WIR with 72 KSN and 73 SNE. This was done to simplify the analysis, and was not recommended for real case analyses. Examining the protein tertiary structure predicted by SCRATCH, we added another group (20 LKI), a member of alpha helix, which made a total of 9 groups [48]. We assigned 87 3-codon DNA 9-mers into 9 FSs according to the outliers. These include 4 WTS (3-codon DNA 9-mer: 0–12) in FS1; 20 LKI (alpha helix1: 13–21) in FS2; 24 STY (22–29) in FS3; 30 VEW (30–32) in FS4; 33 IPY, 61 GPL, and 63 LYY (33–35, 52–65) in FS5; 39 INI and 86 IKE (36–41, 82–86) in FS6; 50 NTA and 51 TAV (42–51) in FS7; 69 WIR, 72 KSN, and 73 SNE (66–73) in FS8; as well as 80 NYL (alpha helix2: 74–81) in FS9 (Figure S5).

Generally, the mean of the correlations of a pair of genera belonging to the same taxonomic group was the highest in each taxonomic group for all 9 FSs (Tables S2 and S3). Table S4 shows the mean and the standard deviation of 9 FSs calculated from the mean of the correlations of a pair of genera belonging to the same taxonomic group in a FS.

The mean of the correlations of a pair of taxonomic groups based on 3-codon usage (left) and the mean based on trimer usage bias (right) are shown in Table S3. Most of them had strong correlations in both alpha helices — FS2 and FS9. This may suggest that roles of amino acids and of codons in alpha helices may be relatively more conserved across taxonomic groups due to functional and structural constraints compared to those in random coils and loops of which flexible structures are equipped for a variety of functions.

### Comparable properties of 25 selected sequences in AMF CDS test set

In order to show that the program provided outliers of 3-codon DNA 9-mers in loading clusters based on 80% components not only in the example sequence but also in all 25 sequences, we included the cluster results of 5 additional query sequences. Genus clusters and loading clusters of the sequences are shown in Tables S5 and S6, respectively. The early diverged bacteria and AMF were often clustered as a sole member or with each other. A great majority of 3-codon DNA 9-mers were grouped together into one major cluster, while outliers were clustered as a sole member or with only one other member.

## Future work

It has been decades since important functions of non-coding RNAs (ncRNAs) were discovered. A previous study has documented that long non-coding RNAs (lncRNAs) play important roles in various cellular processes [51]. Because a large number of lncRNAs contain putative ORFs, it is challenging to distinguish them from protein CDS, especially in AMF CDS database created based on results from a number of gene prediction programs. In the future, we may take a different approach depending on whether a query sequence transcribes either a coding or a non-coding transcript, or both. In addition, we will take into consideration the presence of interaction sites with ncRNAs in a query sequence, because structures required for interactions may impose considerable constraints on codon usages and codon contexts.

Recent studies have documented that codon usage and mRNA structure regulate protein folding [25], [26], [28], [30]. For example, some studies have shown associations between rare codons or double stranded mRNA structures and a decrease of translational speed [26], [30]. Other studies have documented relationships between protein secondary structure and mRNA structure; double stranded mRNA regions tend to have an association with alpha helix and beta strand, while single stranded mRNA regions tend to have an association with random coils [52], [53]. However, the roles of the codons involved in these rules may vary widely across taxonomic groups. Furthermore, while we need defined structures across various taxa, they are mostly from a small number of model organisms. Therefore, it is challenging to study associations between mRNA structures and their corresponding protein structures in metagenome sequencing data. We may be able to improve SeSaMe PS Function by incorporating a new feature that predicts mRNA single and double stranded regions in a query sequence.

## Data availability

SeSaMe PS Function is freely accessible at www.fungalsesame.org.

## CRediT author statement

**Jee Eun Kang:** Conceptualization, Methodology, Software, Validation, Writing - original draft. **Antonio Ciampi:** Supervision, Writing - review & editing. **Mohamed Hijri:** Supervision, Writing - review & editing. All authors read and approved the final manuscript.

## Competing interests

The authors have declared no competing interests.

## References

[1] Hijri M. (2016). Analysis of a large dataset of mycorrhiza inoculation field trials on potato shows highly significant increases in yield. Mycorrhiza.

[2] Roy-Bolduc A., Hijri M. (2011). The use of mycorrhizae to enhance phosphorus uptake: a way out the phosphorus crisis. J Biofertil Biopestic.

[3] Zarik L., Meddich A., Hijri M., Hafidi M., Ouhammou A., Ouahmane L. (2016). Use of arbuscular mycorrhizal fungi to improve the drought tolerance of *Cupressus atlantica* G. C R Biol.

[4] Chanda D., Sharma G.D., Jha D.K., Hijri M. (2014). Associations of arbuscular mycorrhizal (AM) fungi in the phytoremediation of trace metal (TM) contaminated soils. J Res Biol.

[5] Lahlali R., Hijri M. (2010). Screening, identification and evaluation of potential biocontrol fungal endophytes against *Rhizoctonia solani* AG3 on potato plants. FEMS Microbiol Lett.

[6] Iffis B., St-Arnaud M., Hijri M. (2014). Bacteria associated with arbuscular mycorrhizal fungi within roots of plants growing in a soil highly contaminated with aliphatic and aromatic petroleum hydrocarbons. FEMS Microbiol Lett.

[7] Iffis B., St-Arnaud M., Hijri M. (2016). Petroleum hydrocarbon contamination, plant identity and arbuscular mycorrhizal fungal (AMF) community determine assemblages of the AMF spore-associated microbes. Environ Microbiol.

[8] Hassan S.E., St-Arnaud M., Labreque M., Hijri M., Thangadurai D., Busso C.A., Hijri M. (2010). Phytoremediation: biotechnological procedures involving plants and arbuscular mycorrhizal fungi. Mycorrhizal biotechnology.

[9] Hassan S.E., Hijri M., St-Arnaud M. (2013). Effect of arbuscular mycorrhizal fungi on trace metal uptake by sunflower plants grown on cadmium contaminated soil. N Biotechnol.

[10] Hassan SE, Bell TH, Stefani FOP, Denis D, Hijri M, St-Arnaud M., et al. Contrasting the community structure of arbuscular mycorrhizal fungi from hydrocarbon-contaminated and uncontaminated soils following willow (*Salix* spp. L.) planting. PLoS One 2014;9:e102838. 10.1371/journal.pone.0102838 PMC4102571 25032685

[11] Marleau J., Dalpe Y., St-Arnaud M., Hijri M. (2011). Spore development and nuclear inheritance in arbuscular mycorrhizal fungi. BMC Evol Biol.

[12] Hijri M., Redecker D., Petetot J.A.M.C., Voigt K., Wöstemeyer J., Sanders I.R. (2002). Identification and isolation of two ascomycete fungi from spores of the arbuscular mycorrhizal fungus *Scutellospora castanea*. Appl Environ Microbiol.

[13] Cruz A.F., Ishii T. (2012). Arbuscular mycorrhizal fungal spores host bacteria that affect nutrient biodynamics and biocontrol of soil-borne plant pathogens. Biol Open.

[14] Jargeat P., Cosseau C., Ola'h B., Jauneau A., Bonfante P., Batut J. (2004). Isolation, free-living capacities, and genome structure of “*Candidatus* Glomeribacter gigasporarum”, the endocellular bacterium of the mycorrhizal fungus *Gigaspora margarita*. J Bacteriol.

[15] Gulbis N., Robinson-Boyer L., Robinson G. (2013). Studying the microbiome of AMF cultivated *in vitro*. Asp Appl Biol.

[16] Agnolucci M., Battini F., Cristani C., Giovannetti M. (2015). Diverse bacterial communities are recruited on spores of different arbuscular mycorrhizal fungal isolates. Biol Fertil Soils.

[17] Bécard G., Fortin J.A. (1988). Early events of vesicular-arbuscular mycorrhiza formation on Ri T-DNA transformed roots. New Phytol.

[18] Declerck S, Seguin S, Dalpe Y. The monoxenic culture of arbuscular mycorrhizal fungi as a tool for germplasm collections. In: Declerck S, Strullu DG, Fortin JA, editors. *In vitro* culture of mycorrhizas. Berlin Heidelberg: Springer-Verlag; 2005, p. 17–30.

[19] Tisserant E., Malbreil M., Kuo A., Kohler A., Symeonidi A., Balestrini R. (2013). Genome of an arbuscular mycorrhizal fungus provides insight into the oldest plant symbiosis. Proc Natl Acad Sci U S A.

[20] Boon E., Halary S., Bapteste E., Hijri M. (2015). Studying genome heterogeneity within the arbuscular mycorrhizal fungal cytoplasm. Genome Biol Evol.

[21] Lecomte J., St-Arnaud M., Hijri M. (2011). Isolation and identification of soil bacteria growing at the expense of arbuscular mycorrhizal fungi. FEMS Microbiol Lett.

[22] Kang JE, Ciampi A, Hijri M. SeSaMe: metagenome sequence classification of arbuscular mycorrhizal fungi associated microorganisms. Genomics Proteomics Bioinformatics 2020;18:601–612. 10.1016/j.gpb.2018.07.010 PMC8377386 33346086

[23] Berman H.M., Westbrook J., Feng Z., Gilliland G., Bhat T.N., Weissig H. (2000). The Protein Data Bank. Nucleic Acids Res.

[24] Bartoszewski R., Króliczewski J., Piotrowski A., Jasiecka A.J., Bartoszewska S., Vecchio-Pagan B. (2016). Codon bias and the folding dynamics of the cystic fibrosis transmembrane conductance regulator. Cell Mol Biol Lett.

[25] Del Campo C., Bartholomäus A., Fedyunin I., Ignatova Z. (2015). Secondary structure across the bacterial transcriptome reveals versatile roles in mRNA regulation and function. PLoS Genet.

[26] Costafreda M.I., Perez-Rodriguez F.J., D'Andrea L., Guix S., Ribes E., Bosch A. (2014). Hepatitis A virus adaptation to cellular shutoff is driven by dynamic adjustments of codon usage and results in the selection of populations with altered capsids. J Virol.

[27] Komar A.A. (2016). The yin and yang of codon usage. Hum Mol Genet.

[28] Zhao F., Yu C., Liu Y. (2017). Codon usage regulates protein structure and function by affecting translation elongation speed in *Drosophila* cells. Nucleic Acids Res.

[29] McCarthy C., Carrea A., Diambra L. (2017). Bicodon bias can determine the role of synonymous SNPs in human diseases. BMC Genomics.

[30] Yang J. (2017). Does mRNA structure contain genetic information for regulating co-translational protein folding?. Zool Res.

[31] Kashiwagi A., Sugawara R., Sano Tsushima F., Kumagai T., Yomo T. (2014). Contribution of silent mutations to thermal adaptation of RNA bacteriophage Qß. J Virol.

[32] Xu Y., Ma P., Shah P., Rokas A., Liu Y., Johnson C.H. (2013). Non-optimal codon usage is a mechanism to achieve circadian clock conditionality. Nature.

[33] Zhou M., Guo J., Cha J., Chae M., Chen S., Barral J.M. (2013). Non-optimal codon usage affects expression, structure and function of clock protein FRQ. Nature.

[34] Su Y, Jiang XZ, Wu WP, Wang MM, Hamid MI, Xiang MC, et al. Genomic, transcriptomic and proteomic analysis provide insights into the cold adaptation mechanism of the obligate psychrophilic fungus *Mrakia psychrophila*. G3 2016;6:3603–13. 10.1534/g3.116.033308 PMC5100859 27633791

[35] Sanjukta R., Farooqi M.S., Sharma N., Rai A., Mishra D.C., Singh D.P. (2012). Trends in the codon usage patterns of *Chromohalobacter salexigens* genes. Bioinformation.

[36] Bellaousov S., Reuter J.S., Seetin M.G., Mathews D.H. (2013). RNAstructure: web servers for RNA secondary structure prediction and analysis. Nucleic Acids Res.

[37] Defrance M., van Helden J. (2009). Info-gibbs: a motif discovery algorithm that directly optimizes information content during sampling. Bioinformatics.

[38] Solovyev V., Salamov A., Li R.W. (2011). Automatic annotation of microbial genomes and metagenomic sequences. Metagenomics and its applications in agriculture, biomedicine and environmental studies.

[39] Lee T.Y., Chang W.C., Hsu J., Chang T.H., Shien D.M. (2012). GPMiner: an integrated system for mining combinatorial cis-regulatory elements in mammalian gene group. BMC Genomics.

[40] Yan J., Kurgan L. (2017). DRNApred, fast sequence-based method that accurately predicts and discriminates DNA- and RNA-binding residues. Nucleic Acids Res.

[41] Lokanath N.K., Pampa K.J., Takio K., Kunishima N. (2008). Structures of dimeric nonstandard nucleotide triphosphate pyrophosphatase from *Pyrococcus horikoshii* OT3: functional significance of interprotomer conformational changes. J Mol Biol.

[42] Qian C., Li S., Jakoncic J., Zeng L., Walsh M.J., Zhou M.-M. (2008). Structure and hemimethylated CpG binding of the SRA domain from human UHRF1. J Biol Chem.

[43] Nikolovska-Coleska Z., Meagher J.L., Jiang S., Yang C.Y., Qiu S., Roller P.P. (2008). Interaction of a cyclic, bivalent Smac mimetic with the X-linked inhibitor of apoptosis protein. Biochemistry.

[44] Tucker T.J., Sisko J.T., Tynebor R.M., Williams T.M., Felock P.J., Flynn J.A. (2008). Discovery of 3-{5-[(6-Amino-1H-pyrazolo[3,4-b]pyridine-3-yl)methoxy]-2-chlorophenoxy}-5-chlorobenzonitrile (MK-4965): a potent, orally bioavailable HIV-1 non-nucleoside reverse transcriptase inhibitor with improved potency against key mutant viruses. J Med Chem.

[45] Furnham N., Holliday G.L., de Beer T.A.P., Jacobsen J.O.B., Pearson W.R., Thornton J.M. (2014). The Catalytic Site Atlas 2.0: cataloging catalytic sites and residues identified in enzymes. Nucleic Acids Res.

[46] Huang Z., Zhu L., Cao Y., Wu G., Liu X., Chen Y. (2011). ASD: a comprehensive database of allosteric proteins and modulators. Nucleic Acids Res.

[47] Abdi H., Williams L.J. (2010). Principal component analysis. Wiley Interdiscip Rev Comput Stat.

[48] Cheng J., Randall A.Z., Sweredoski M.J., Baldi P. (2005). SCRATCH: a protein structure and structural feature prediction server. Nucleic Acids Res.

[49] D'Souza D.J., Kool E.T. (1992). Strong binding of single-stranded DNA by stem-loop oligonucleotides. J Biomol Struct Dyn.

[50] Tan D., Marzluff W.F., Dominski Z., Tong L. (2013). Structure of histone mRNA stem-loop, human stem-loop binding protein and 3'hExo ternary complex. Science.

[51] Achawanantakun R., Chen J., Sun Y., Zhang Y. (2015). LncRNA-ID: long non-coding RNA identification using balanced random forests. Bioinformatics.

[52] Jia M., Luo L., Liu C. (2004). Statistical correlation between protein secondary structure and messenger RNA stem-loop structure. Biopolymers.

[53] Zhang J., Gu B.H., Peng S.L., Liu C.Q. (1998). Distributions of triplet codons in messenger RNA secondary structures. Zool Res.

