# Supplementary material for: SeSaMe PS Function: Functional Analysis of the Whole Metagenome Sequencing Data of the Arbuscular Mycorrhizal Fungi
Source: Genomics Proteomics Bioinformatics. 2020 Dec 18;18(5):613–23. doi: 10.1016/j.gpb.2018.07.011 (PMC8377382; doi:10.1016/j.gpb.2018.07.011)
Supplement: Supplementary Table S5 [file mmc5.doc]

***Table S5 Genus clusters of five additional sequences***

|  | **seq252** | **seq284** | **seq337** | **seq475** | **seq528** |
| --- | --- | --- | --- | --- | --- |
| **Cluster 0** | 0,1,2,4,5,9,10,16,17,19,21,22,23,24,25,26,29,30,34,35,36,37,40,41,42,43,44,49,51 | 4,5,17,22,34,37,41,43,44 | 1,4,5,9,10,16,19,21,22,23,24,25,26,30,34,35,36,37,40,41,42,43,44,51 | 7,13,28,33 | 1,2,4,5,8,9,10,16,19,21,22,23,24,25,26,30,34,35,36,37,40,41,42,43,44,51 |
| **Cluster 1** | 32,39 | 3,12,20,31,38 | 12,20 | 11,45 | 11,45 |
| **Cluster 2** | 38 | 7,13,27,32,39,47,50,52,53 | 13,31 | 4,5,9,10,16,19,22,23,24,25,30,34,35,37,41,42,43,44 | 6,12 |
| **Cluster 3** | 6,20 | 11,45 | 38,45 | 27,29,39,47,48,50,52,53 | 3,20 |
| **Cluster 4** | 7,8,14,15,18,46,47,48,52,53 | 6,33 | 27,33 | 6,12 | 27,33 |
| **Cluster 5** | 11,45 | 9 | 7,8,14,15,29,39,47,50,52,53 | 0,1,2,18,21,26,36,40 | 28,32,39 |
| **Cluster 6** | 33,50 | 1,2,21,26,36,40 | 0,2,17,18,46,48,49 | 3,20 | 38 |
| **Cluster 7** | 12 | 28,29 | 3,6 | 38 | 7,13,31 |
| **Cluster 8** | 3,13 | 0,8,14,15,18,46,48,49,51 | 11 | 8,14,15,17,46,49,51 | 14,15,18,29,47,48,50,52,53 |
| **Cluster 9** | 27,28,31 | 10,16,19,23,24,25,30,35,42 | 28,32 | 31,32 | 0,17,46,49 |

*Note*: The numbers in the table stand for the genera: 0:*Acidithiobacillus*, 1:*Acidobacterium*, 2:*Agrobacterium*, 3:*Anabaena*, 4:*Azorhizobiu*m, 5:*Azotobacter*, 6:*Bacillus*, 7:*Bdellovibrio*, 8:*Beijerinckia*, 9:*Bradyrhizobium*, 10:*Caulobacter*, 11:*Clostridium*, 12:*Cyanobacterium*, 13:*Desulfotomaculu*m, 14:*Desulfovibrio*, 15:*Erwini*a, 16:*Frankia*, 17:*Geobacter*, 18:*Klebsiella*, 19:*Kocuria*, 20:*Leuconostoc*, 21:*Mesorhizobium*, 22:*Methylococcus*, 23:*Microbacterium*, 24:*Micrococcus*, 25:*Myxococcus*, 26:*Nitrobacter*, 27:*Nitrosococcus*, 28:*Nitrosomonas*, 29:*Nitrosospira*, 30:*Nocardia*, 31:*Nostoc*, 32:*Oscillatoria*, 33:*Pseudanabaena*, 34:*Pseudomonas*, 35:*Pseudonocardia*, 36:*Rhizobium*, 37:*Rhodobacter*, 38:*Rickettsia*, 39:*Shewanella*, 40:*Sinorhizobium*, 41:*Sphingomonas*, 42:*Streptomyces*, 43:*Variovorax*, 44:*Xanthomonas*, 45:AMF, 46:*Aspergillus*, 47:*Cenococcum*, 48:*Cryptococcus*, 49:*Mycosphaerella*, 50:*Oidiodendron*, 51:*Phanerochaete*, 52:*Scleroderma*, 53:*Sebacina*.
