# Supplementary material for: SeSaMe PS Function: Functional Analysis of the Whole Metagenome Sequencing Data of the Arbuscular Mycorrhizal Fungi
Source: Genomics Proteomics Bioinformatics. 2020 Dec 18;18(5):613–23. doi: 10.1016/j.gpb.2018.07.011 (PMC8377382; doi:10.1016/j.gpb.2018.07.011)
Supplement: Supplementary Table S6 [file mmc6.doc]

***Table S6 Loading clusters of five additional sequences***

| **seq252** | **seq284** | **seq337** | **seq475** | **seq528** |
| --- | --- | --- | --- | --- |
| Cluster 0  69_JEJ_VNL_GTGAATTTG | Cluster 0  74_HGA_PGK_CCAGGAAAG | Cluster 0  12_KKJ_YFL_TATTTTCTC | Cluster 0  51_DDD_SSS_AGTTCGAGT | Cluster 0  7_AGJ_KGA_AAAGGAGCA |
| Cluster 1  59_EKA_NWK_AATTGGAAA | Cluster 1  36_JJD_IVT_ATAGTGACT | Cluster 1  Major cluster | Cluster 1  45_DGD_SGT_TCAGGTACT | Cluster 1  Major cluster |
| Cluster 2  34_AJE_RAN_AGAGCAAAT | Cluster 2  27_JHJ_LPI_TTGCCGATT | Cluster 2  8_DDK_TSF_ACATCTTTT | Cluster 2  43_GDD_GTS_GGTACTTCA | Cluster 2  24_AEJ_KNA_AAAAATGCG |
| Cluster 3  30_GAJ_GKV_GGTAAAGTG | Cluster 3  52_CHJ_EPI_GAACCAATC,  64_JJD_ILS_ATTTTGTCA | Cluster 3  46_ICJ_MDA_ATGGATGCA | Cluster 3  24_EAD_NKS_AATAAATCA,  56_EAK_QKY_CAAAAATAT | Cluster 3  31_JDA_ASK_GCCTCGAAA |
| Cluster 4  Major cluster | Cluster 4  1_GEH_GNP_GGTAACCCA | Cluster 4  40_AKC_KFE_AAATTTGAG | Cluster 4  11_CAJ_DKL_GACAAATTA,  26_DJC_SAE_TCAGCTGAA | Cluster 4  17_AEK_RNF_AGAAATTTT |
| Cluster 5  37_KKC_WFD_TGGTTTGAT | Cluster 5  Major cluster | Cluster 5  52_AJE_KLQ_AAACTTCAA | Cluster 5  Major cluster | Cluster 5  25_EJA_NAK_AATGCGAAA |
| Cluster 6  1_BDK_HTF_CATACATTC | Cluster 6  43_AJA_KLR_AAACTACGC | Cluster 6  27_AEG_RNG_CGTAATGGG | Cluster 6  40_AJA_RLR_AGATTACGA | Cluster 6  20_EKK_NYY_AATTATTAT |
| Cluster 7  70_EJA_NLK_AATTTGAAA,  71_JAD_LKS_TTGAAAAGT | Cluster 7 26_CJH_ELP_GAATTGCCG | Cluster 7  2_JJD_LVS_TTGGTATCA | Cluster 7  27_JCE_AEQ_GCTGAACAG | Cluster 7  36_AJH_KAP_AAAGCTCCA |
| Cluster 8  26_ACC_KDE_AAAGATGAG | Cluster 8 17_AAE_RRN_CGTAGAAAC | Cluster 8  5_DAK_TKY_ACTAAGTAT | Cluster 8  28_CEG_EQG_GAACAGGGA | Cluster 8  30_CJD_DAS_GATGCCTCG |
| Cluster 9  18_JKC_LYD_TTGTATGAC | Cluster 9 37_JDC_VTE_GTGACTGAA | Cluster 9  22_EKJ_QFI_CAATTCATA | Cluster 9  14_CCD_DDT_GACGACACA | Cluster 9  1_CDJ_ESL_GAATCTCTT |
| Cluster 10  31_AJK_KVY_AAAGTGTAT | Cluster 10 45_AKA_RYR_CGCTATAGA | Cluster 10  4_DDA_STK_TCAACTAAG | Cluster 10  33_CJA_EAR_GAAGCTAGG | Cluster 10  11_JJJ_LLI_TTATTAATC,  34_JDA_ITK_ATTACAAAA |
| Cluster 11  40_GEJ_GNI_GGGAACATA | Cluster 11 57_KIE_YMQ_TATATGCAA | Cluster 11  53_JEJ_LQI_CTTCAAATA | Cluster 11  29_EGE_QGN_CAGGGAAAT | Cluster 11  5_JDA_ATK_GCTACAAAA,  18_EKE_NFN_AATTTTAAT |
| Cluster 12  36_EKK_NWF_AATTGGTTT | Cluster 12 78_KJI_YLM_TATCTCATG | Cluster 12  7_KDD_YTS_TATACATCT,  35_KCH_YEP_TATGAACCA,  36_CHJ_EPI_GAACCAATT,  37_HJA_PIK_CCAATTAAA | Cluster 12  37_DKK_SFY_AGTTTTTAT | Cluster 12  13_JED_INT_ATCAACACT |

*Note*: The major cluster contains the rest of the 3-codon DNA 9-mers that were omitted from the table. seq252:GCACATACATTCTATGAAGTAAATAATGCATTAGAATGGATACCTTATGATAAATTGTATGACATTAAATATATTACGAAAGATGAGTTAGGTAAAGTGTATAGAGCAAATTGGTTTGATGGGAACATAATTGATAAATATTATAGTTATAATTATTGGGGTGATGTATTAAAACATAATTGGAAAAGAAACTATCCTAATATGTTTGTGAATTTGAAAAGTTTAAATTCTCCAAATGATCTTAC ;

seq284:CCAATGGTAACCCAAATGGAAATGATAATGGTAATGGCAATGGTACAGAACGACGTAGAAACGTAGAAGATCTTTATTCTGAATTGCCGATTGATAGTAAAACTAAGGAAATAGTGACTGAAGTTAATGCAAAACTACGCTATAGATATGTAAATATGGAACCAATCAAGCTTTATATGCAAGTTTGCCAATTTATTTTGTCATTATTTCCTGATGTACCGGATCCAGGAAAGTTATATCTCATGTTTCCGGATGGTAAAA ;

seq337:TATTTATTTGGTATCAACTAAGTATACATCTTTTTTATATTTTCTCTTTCCAAAATTAACAAATTTACAATTCATAAGAATACGTAATGGGGATAATATTAATAATTATGAACCAATTAAAAAATTTGAGGAATACGCAATGGATGCAAGTTATTATAAACTTCAAATACTTGAGT ;

seq475:AGCTCAATACAATCTTGGAGTTATTTATGAAACTGACAAATTAGACGACACAATTGCAGCACTGTATTGGTATAATAAATCAGCTGAACAGGGAAATCATGAAGCTAGGGAAAGTTTTTATAGATTACGAGGTACTTCAGGTACTAAGACTGTTAGTTCGAGTAGTATACAAAAATATGGTTCTATGGGTAT ;

seq528:CTTGAATCTCTTCTTGCTACAAAAGGAGCAGAGTTATTAATCAACACTTTAAGAAATTTTAATTATTATAAAAAAAATGCGAAAGAACAAGATGCCTCGAAAATTACAAAAGCTCCAAAAATTAAAAAAGAAATGAGTAAAATTAAGTGGTCACAAATT.
